# Pegylated interferon for treating severe recurrent respiratory papillomatosis in a child: case report

**DOI:** 10.1590/1516-3180.2017.0031240317

**Published:** 2017-09-18

**Authors:** Rebecca Maunsell, Maria Angela Bellomo-Brandão

**Affiliations:** I Medical Doctor and Associate Professor, Department of Otolaryngology, Universidade Estadual de Campinas (UNICAMP), Campinas (SP), Brazil.; II Medical Doctor and Associate Professor, Department of Pediatrics, Universidade Estadual de Campinas (UNICAMP), Campinas (SP), Brazil.

**Keywords:** Papillomatosis, Respiratory, Peginterferon, Child

## Abstract

**CONTEXT:**

Recurrent respiratory papillomatosis (RRP) is the most common laryngeal tumor. During childhood, it may present in extremely severe forms defined by the need for frequent surgical procedures to relieve respiratory distress and/or involvement of extralaryngeal sites such as lung involvement. Adjuvant therapies are indicated in these cases and interferon is one of the options. Pegylated interferon is more effective than conventional alpha interferon and, given its reported results in relation to treating hepatitis C over the past decade, we hypothesized that this might be more effective than conventional interferon also for treating respiratory papillomatosis. Use of a treatment strategy that eliminates the need for general anesthesia is particularly appealing, yet obtaining approval for use of medications that are not currently used for this purpose is challenging.

**CASE REPORT:**

We report the case of a child with severe RRP that had been followed for the preceding six years, who was treated with pegylated interferon after failure of other adjuvant therapies. There was noticeable improvement in the frequency of surgical procedures, which was regarded very receptively, considering the child’s history and previous response to other therapies.

**CONCLUSION:**

Pegylated interferon may be a good option for diminishing the need for surgical intervention in severe cases of recurrent respiratory papillomatosis.

## INTRODUCTION

Recurrent respiratory papillomatosis (RRP) is the most common benign tumor of the larynx and occurs both in adults and in children. An estimated 4.5 cases per 100,000 occur in the United States.^1^ Many medical and surgical treatments have been described but the cure remains unknown.

When the disease initially presents before the age of three years, the need for frequent surgical intervention is more common^1^ and acute episodes of respiratory obstruction can be quite dramatic and even fatal. This gives rise to an emotional burden both for the child and for the family involved. Lesions that occur prematurely under the age of 12 months and infection with human papilloma virus type 11 are frequently associated with worse prognosis and greater severity of clinical presention.^2^

Surgical interventions are the only way to secure a patent airway and various instruments can be used depending on the surgeon’s experience and the available technology. Cold instrumentation, carbon dioxide laser and microdebridement are the methods most frequently reported. Repeated surgical intervention facilitates disease dissemination to other sites of the airway and increases the risk of definitive scarring of the larynx, with significant sequelae for the voice. Adjuvant treatments may be needed in up to 20% of the cases.^1^^,^^2^

The recommended indications for adjuvant therapy are: the need for more than four surgical procedures per year; rapid growth of papillomata with airway compromise; or distal multisite spreading of disease.^1^ Extralaryngeal involvement may occur in up to 30% of the cases,^3^ with lesions occurring in the oral cavity, trachea, bronchi and esophagus. Malignant transformation, although rare, may occur in around 1.6-2% of cases.^3^

The adjuvant treatments for RRP include use of cidofovir, cis-retinoic acid, ribavirin, indole-3-carbinol, human papillomavirus (HPV) vaccine, bevacizumab, photodynamic therapy, celecoxib and interferon.^4^^,^^5^ The most commonly used adjuvant treatment is cidofovir,^4^^,^^5^ and this involves intralesional application of medication that is performed under general anesthesia. Thus, despite improving the disease, it does not eliminate the need for hospitalization and use of anesthesia for microlaryngoscopy. Unfortunately, there is a general lack of evidence to support these therapies when not in the context of clinical research.

Alpha-interferon was the first adjuvant treatment used to treat respiratory papillomatosis, but the evidence has failed to support its use, based on limited case series reports and a few controlled studies that lacked sustainable results, despite the initial improvement observed when alpha-interferon therapy was combined with surgical removal of tumors.^6^ Systemic side-effects and high costs are also a disadvantage of interferon.

Use of adjuvant treatments that avoid the need for surgical intervention and anesthesia is of particular interest, since repeated procedures and exposure to general anesthesia are a burden for these children and their families.

The advantage of pegylated interferon (PEG-IFN) over conventional interferon has been proven through its use for treating hepatitis C. This benefit is due mostly to its sustained levels in plasma, which translates into better treatment results. Since 2001,^7^ significant improvement in treating hepatitis C through using PEG-IFN, in comparison with conventional alpha-interferon, has been continually reported. Use of PEG-IFN has revolutionized the treatment of hepatitis C worldwide.^7^ There are no reports of use of pegylated interferon as an adjuvant treatment for recurrent respiratory papillomatosis in children.

The present study is a case report on a pediatric patient with recurrent respiratory papillomatosis who was treated with both surgical resection and adjuvant therapies, including use of PEG-IFN, with noticeable improvement in recurrence. Following the case report, we present a brief discussion and a systematic search of data in the PubMed, Cochrane, LILACS and Embase databases (Table 1). The articles included were either clinical trials on alpha-interferon or case reports on PEG-IFN. Duplicated studies and those that did not refer specifically to respiratory papillomatosis were excluded.

**Table 1.** Description of yearly follow-up: number of procedures, instrumentation used, adjuvant treatments, presence of tracheostomy and interval between surgical procedures (mean in days)

	Year 1	Year 2	Year 3	Year 4	Year 5	Year 6
Number of procedures	16	8	6	4	8	5
Surgical instrumentation	cold/laser	cold/mdb	mdb	mdb	mdb	mdb
Adjuvant treatment	cidofovir	bevacizumab	none	none	none	PEG-IFN
Tracheostomy	yes	yes	yes	yes	no	no
Interval between procedures (days)	22.3	45.5	61.3	83.2	43.8	77.2

mdb = microdebridement; PEG-IFN = pegylated interferon.

## CASE REPORT

Institutional ethics committee approval was obtained (Ethics Committee of the School of Medical Sciences, State University of Campinas, UNICAMP) under protocol number 020/2016, subsequent to obtaining signed parental consent for submission of this communication.

**Figure 1.**
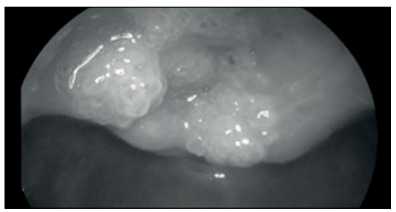
Laryngoscopic view of the laryngeal inlet obstructed by profuse papilloma lesions.

A male child aged 14 months presenting recurrent episodes of suspected laryngitis and upper airway obstruction was diagnosed with laryngeal papillomatosis after nasofibroscopy revealed typical papillomatous lesions of the larynx (Figure 1).

**Figure 2.**
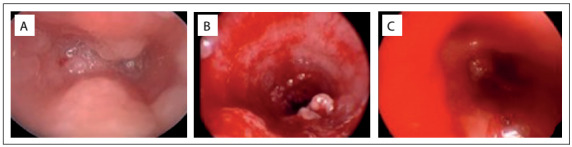
Laryngoscopic view of the upper trachea with papilloma lesions surrounding supra-stomal trachea (A), middle trachea (B) and bronchi (C).

The diagnosis was confirmed through a biopsy specimen and hybridization confirmed the presence of infection with HPV type 11. Initially, the treatment consisted of multiple airway procedures to remove obstructive lesions from the airway. The criterion for surgical removal of lesions was respiratory distress. After eight surgical procedures, at the age of 17 months the child underwent tracheostomy and adjuvant treatment with cidofovir was introduced, following parental informed consent. At this time, the lesions involved both of the vocal cords and also the laryngeal ventricles, false vocal cords, epiglottis and peristomal trachea (Figure 2a and b).

Intralesional applications of cidofovir were performed under general anesthesia and were scheduled every 2 to 4 weeks but no sustainable control of disease was achieved, with respiratory distress occurring repeatedly, three weeks after the application. Progression of papilloma lesions to the lower airway was also observed (Figure 2c) during the course of cidofovir treatment, which was halted after 12 applications.

**Figure 3.**
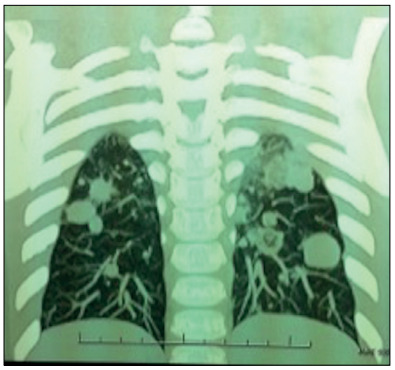
First pulmonary computed tomography scan showing numerous bilateral solid nodules, in the patient at the age of three years.

A computed tomography (CT) scan was performed after use of cidofovir had been halted and after observation of papilloma lesions in the lower airway. It revealed multiple pulmonary nodules ranging from 3 mm to 14 mm in size; cavitary lesions with thick, irregular walls; multiple diffuse small centrilobular nodules; and bilateral hilar and right lower paratracheal and subcarinal lymphadenopathy (Figure 3). A biopsy on one of the pulmonary nodules confirmed the diagnosis of pulmonary papillomatosis.

A trial using intralesional bevacizumab was attempted, but was also halted after three applications, since no noticeable improvement of recurrence rates was achieved and the patient presented moderate hair loss.

**Figure 4.**
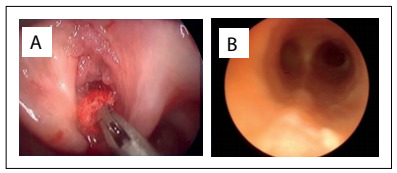
View of the larynx (A) and lower airway (B) during year 5.

Up to the age of 5 years, the tracheostomy helped alleviate the patient’s respiratory distress. The intervals between surgical procedures were as long as 83 days during the fourth year of follow-up (Table 1). Just before the patient reached six years of age, although the recurrence rates were still high, the significant growth of the airway allowed decannulation. After this, without the tracheostomy, deobstruction of the airway was required almost on a monthly basis (year 5 in Table 1) with profuse lesions involving the laryngeal inlet (Figure 4a), despite progressive improvement of the lesions in the lower airway (Figure 4b).

A six-month treatment with PEG-IFN α2 β IFN-α2b (1.0 μg/kg/wk) was proposed in an attempt to increase the intervals between surgical procedures. This was started after receiving informed parental consent. The child was six years old at the time. The detailed yearly follow-up of the child can be seen in Figure 2.

Subcutaneous injections of PEG-IFN (1 μg/kg) were given once a week. Fever, headaches and general flu-like symptoms were observed after the first applications during the initial 48 hours but were well managed with the usual antipyretic drugs. Complete blood count, alanine aminotransferase (ALT), aspartate aminotransferase (AST), alkaline phosphatase (ALP), gamma-glutamyl-transferase (GGT), albumin, international normalized ratio (INR), thyroxine (T4) and thyroid-stimulating hormone (TSH) were monitored monthly.

For more than three consecutive months, for the first time, surgical intervention was not needed. This interval between procedures had not been seen, even when the child still had a tracheostomy. After 102 days, surgery was indicated, for the first time, not due to respiratory distress or stridor but because of vocal demands and progressive moderate to severe dysphonia, which was a hazard at school.

**Figure 5.**
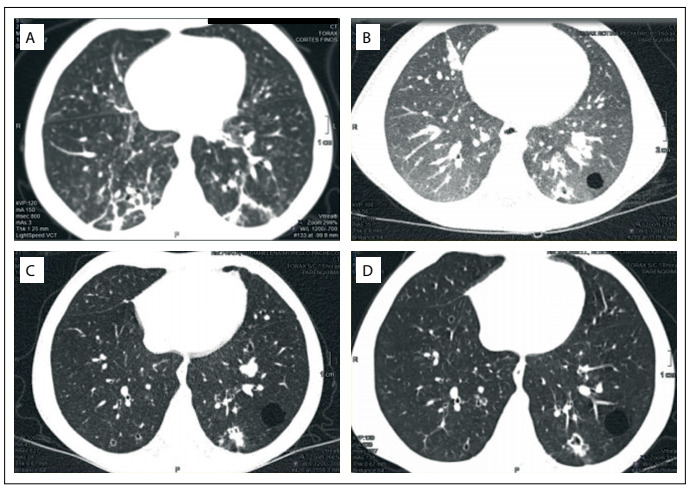
Computed tomography scans in year 3 (A), year 4 (B), year 5 (C) and year 6 (D).

Unfortunately, after a three-month treatment, a threefold elevation in liver transaminase levels was noted and treatment was halted. Despite withdrawing the treatment, the mean interval between procedures improved considerably over the months following PEG-IFN treatment (year 6), in comparison with the previous year (year 5). CT scans performed at yearly intervals showed improvement in the number of solid nodules, particularly in years 5 and 6, in comparison with years 3 and 4 (Figure 5).

Throughout the years of follow-up, the child has maintained mild to moderate and sometimes severe dysphonia, depending on the progressive and intermittent infiltration of the vocal cords with papilloma lesions, but also secondary to the vocal cord scarring that has inevitably occurred. Pulmonary function has remained stable, despite persistent pulmonary lesions. Spirometry performed during follow-up in year 5 was normal.

## DISCUSSION

Pegylated interferon has proven to be significantly more effective for treating hepatitis C than conventional alpha-interferon.^7^^,^^8^ It is only natural to hypothesize that the pegylated form of interferon might be also more effective for treating respiratory papillomatosis than alpha-interferon.

Alpha-interferon is an immunomodulatory cytokine with antiviral and antiproliferative properties and was the first widely used adjuvant drug for treating RRP, with diverse results ranging from better prognosis to absence of response.^6^^,^^9^ The evidence has failed to support its use, since the reports have mostly been based on case reports and clinical trials have shown varied results with limited follow-up.^6^^,^^9^^,^^10^

The association between interferon and polyethylene glycol, which is called pegylated interferon, increases the half-life of the drug, eliminates immunogenicity and improves the pharmacokinetics of the protein, such that it can be administered once a week. It potentially has greater antiviral activity than conventional alpha-interferon.^8^ Since it was first introduced for treating hepatitis C, there has been a complete change of scenario for patients infected with the hepatitis C virus worldwide.

Regarding use of alpha-interferon for treating respiratory papillomatosis, very few clinical trials could be found for this review (Table 2).

**Table 2.** Database search results regarding reports on papillomatosis treated with pegylated interferon (PEG-IFN). Search performed on January 20, 2017

Database	Search strategy	Articles found	Articles included
MEDLINE	(papillomatosis) AND (pegylated interferon) OR (peginterferon)	4	2
Cochrane Library	(papillomatosis) AND (pegylated interferon) OR (peginterferon)	0	0
	(interferon) AND (papillomatosis)	8	3
LILACS	(papillomatosis) AND (pegylated interferon) OR (peginterferon)	0	0
	(papillomatosis) AND (interferon)	13	0
Embase	(papillomatosis) AND (pegylated interferon)	2	1
	(papillomatosis) AND (peginterferon)	10	0

The most relevant clinical trial found in the Cochrane database is the one by Healy et al.^6^ Their study consists of a randomized trial on 123 patients. Patients were assigned either to treatment with interferon and surgery or to surgery alone. They found that the papillomata growth rate was significantly reduced in the group receiving alpha-interferon over the first six months of the treatment. Nevertheless, in the second six months, there was no statistical difference among the groups. The authors concluded that interferon treatment was effective but could not be sustained. The second most relevant study consisted of a randomized crossover trial^9^ describing 66 patients who were treated with interferon. Half of the patients were treated from the beginning of the study period and half waited six months to begin treatment lasting for the same length of time. Comparison of the groups during the first six months showed that there was better control over the disease in the group that underwent interferon therapy, with eight remissions occurring in this group, compared with none in the observation-alone group. Benjamin et al.^10^ conducted a crossover trial on 10 patients and also reported that there was an apparent benefit from using interferon in association with surgical resection, in comparison with surgical resection alone. Complete or partial remission was seen in six of their 10 patients. Complete remission was sustained for two years. It is important to note that these clinical trials were all reported before pegylated interferon became available and therefore before any of the papers published by hepatologists or infectologists that demonstrated improvement in treatment of hepatitis C virus infection in comparison with conventional alpha-interferon.

The only published article describing a series of patients with respiratory papillomatosis who were treated with pegylated interferon reported on 11 adult patients who received PEG-IFN α2a.^11^ They were treated for six months with weekly subcutaneous injections of 180 μg and in the third month were also given granulocyte-monocyte colony-stimulating factor. The authors reported that a significant improvement in voice quality and a reduction in the number of surgical interventions were achieved after a six-month treatment with a 12-month follow-up period.^11^ Another single case report on a 73-year-old patient with longstanding juvenile respiratory papillomatosis described the use of PEG-IFN in an attempt at adjuvant treatment, among other treatments, in the patient’s final years. The patient eventually succumbed to the disease following malignant transformation.^12^

The case that we described here was one with a dramatic burden on the family, since surgical debulking to restore airway patency was required every month, following decannulation. Early pulmonary involvement and progression was also a concern, and these were criteria for adjuvant treatment. In the years without tracheostomy (years 5 and 6), upper airway obstruction was inevitably potentially more symptomatic, with the possibility of a fatal outcome. However, there was a noticeable increase in the interval free from surgical interventions, from 43.8 days before treatment was needed to 77.2 days. This coincided with the pegylated interferon treatment.

Unfortunately, treatment had to be halted after three months, despite the initial plan for a six-month treatment period, due to significant elevation in transaminase levels. It is possible that a longer-lasting response would have been seen with six months of treatment. Nonetheless, one year after halting the treatment, the intervals between procedures were still longer than they were the year before.

This is the first report of use of pegylated interferon for recurrent respiratory papillomatosis in a child. Use of medication for off-label purposes is always a difficult decision. Thus, case reports on extremely severe cases such as the one described here may be of great help for colleagues caring for similar cases. Although a single case report is insufficient to determine whether the improvement can be attributed solely to adjuvant treatment and not the actual natural history of this disease, the difference in this particular case, from before to after interferon treatment, was quite striking in the eyes of the medical team that has closely followed this case for over five years.

Future studies might prove pegylated interferon to be more effective than traditional alpha-interferon, as has been demonstrated with regard to treating hepatitis C.

## CONCLUSION

Pegylated interferon may be a good option for diminishing the need for surgical intervention in severe cases of RRP.
